# Supplementary material for: Identification of 15 T Cell Restricted Genes Evaluates T Cell Infiltration of Human Healthy Tissues and Cancers and Shows Prognostic and Predictive Potential
Source: Int J Mol Sci. 2019 Oct 22;20(20):5242. doi: 10.3390/ijms20205242 (PMC6829269; doi:10.3390/ijms20205242)
Supplement: Supplementary file 1 [file ijms-20-05242-s001.zip › ijms-610023-supplymentary2/Figure S1 IJMS.pdf]

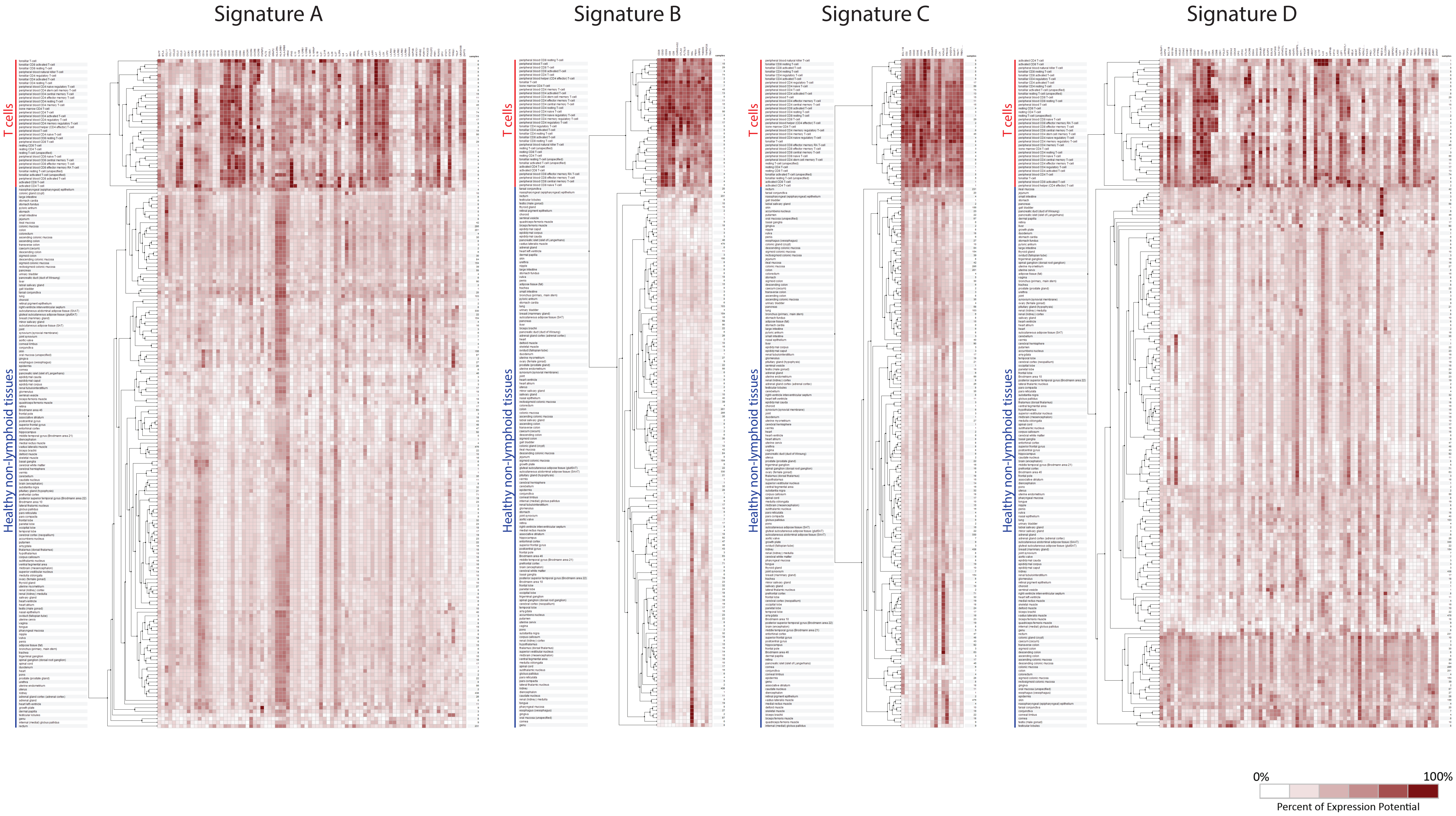

Figure S1: Expression of genes included in previously published T cell signatures

Expression of the genes included in the signatures reported by Angelova et al. (signature A), Becht et al. (signature B), Bindea et al. (signature C) and Zhong et al. (signature D) was evaluated in non-lymphoid tissues from healthy human donors by using the hierarchical clustering tool (Euclidean distance and optimal leaf-ordering) of the Genevestigator suite. For comparison, the expression of the same genes by untouched and activated T cells purified from different human sources was tested. Please note that the order in which healthy non-lymphoid tissues and T cells are presented is different for signatures A-D as a result of hierarchical clusterization.
