# Supplementary material for: Identification of 15 T Cell Restricted Genes Evaluates T Cell Infiltration of Human Healthy Tissues and Cancers and Shows Prognostic and Predictive Potential
Source: Int J Mol Sci. 2019 Oct 22;20(20):5242. doi: 10.3390/ijms20205242 (PMC6829269; doi:10.3390/ijms20205242)
Supplement: Supplementary file 1 [file ijms-20-05242-s001.zip › ijms-610023-supplymentary2/Figure S10 IJMS.pdf]

A

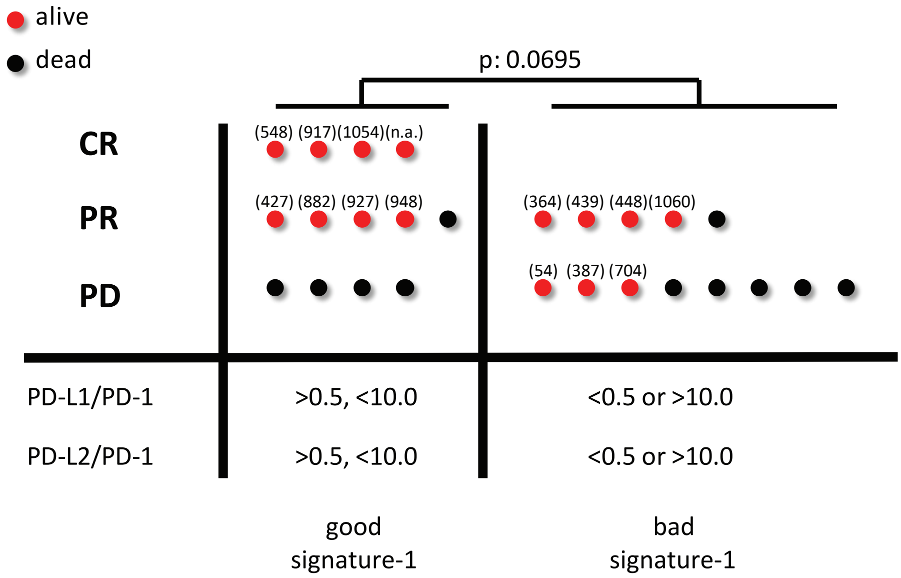

B

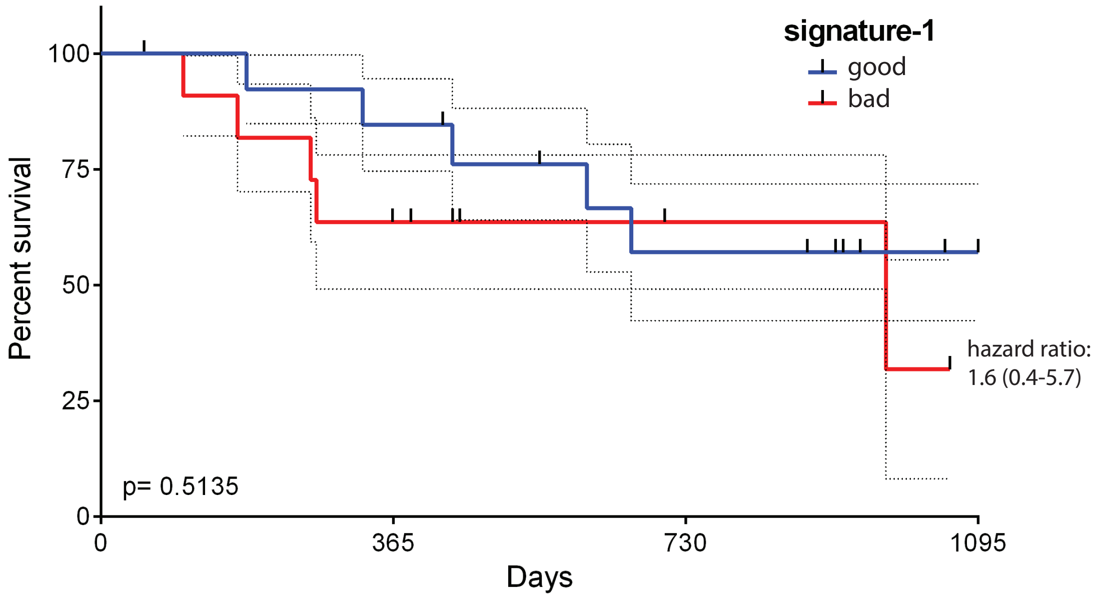

Figure S10: PD-1 ligand/PD-1 ratio as a poor indicator of response to nivolumab treatment

(A) The ligand/receptor ratio in melanoma specimens (Table S11) was evaluated, and patients were ranked into two groups: the good signature-1 group (PD-L1/PD-1 and PD-L2/PD-1 ratio between 0.5 and 10) and the bad signature-1 group (PD-L1/PD-1 and/or PD-L2/PD-1 ratio <0.5 or >10). Dots represent single patients (the red dots indicate living patients and the black dots indicate dead patients) classified according to their response to the treatment (complete response, CR; partial response, PR; progressive disease, PD). The response of the patients in the good and the bad signature-1 groups was not significantly different according to the results of the contingency chi-square test.
(B) The Kaplan-Meier curve for the good signature-1 group (blue line) and the bad signature-1 group (red line) is shown. The survival of the patients in the good and the bad signature-1 groups was not significantly different according to the results of the log-rank Mantel-Cox test. The hazard ratio (95%CI) was calculated with the log-rank method and is shown.
