# Supplementary material for: Identification of 15 T Cell Restricted Genes Evaluates T Cell Infiltration of Human Healthy Tissues and Cancers and Shows Prognostic and Predictive Potential
Source: Int J Mol Sci. 2019 Oct 22;20(20):5242. doi: 10.3390/ijms20205242 (PMC6829269; doi:10.3390/ijms20205242)
Supplement: Supplementary file 1 [file ijms-20-05242-s001.zip › ijms-610023-supplymentary2/Figure S11 IJMS.pdf]

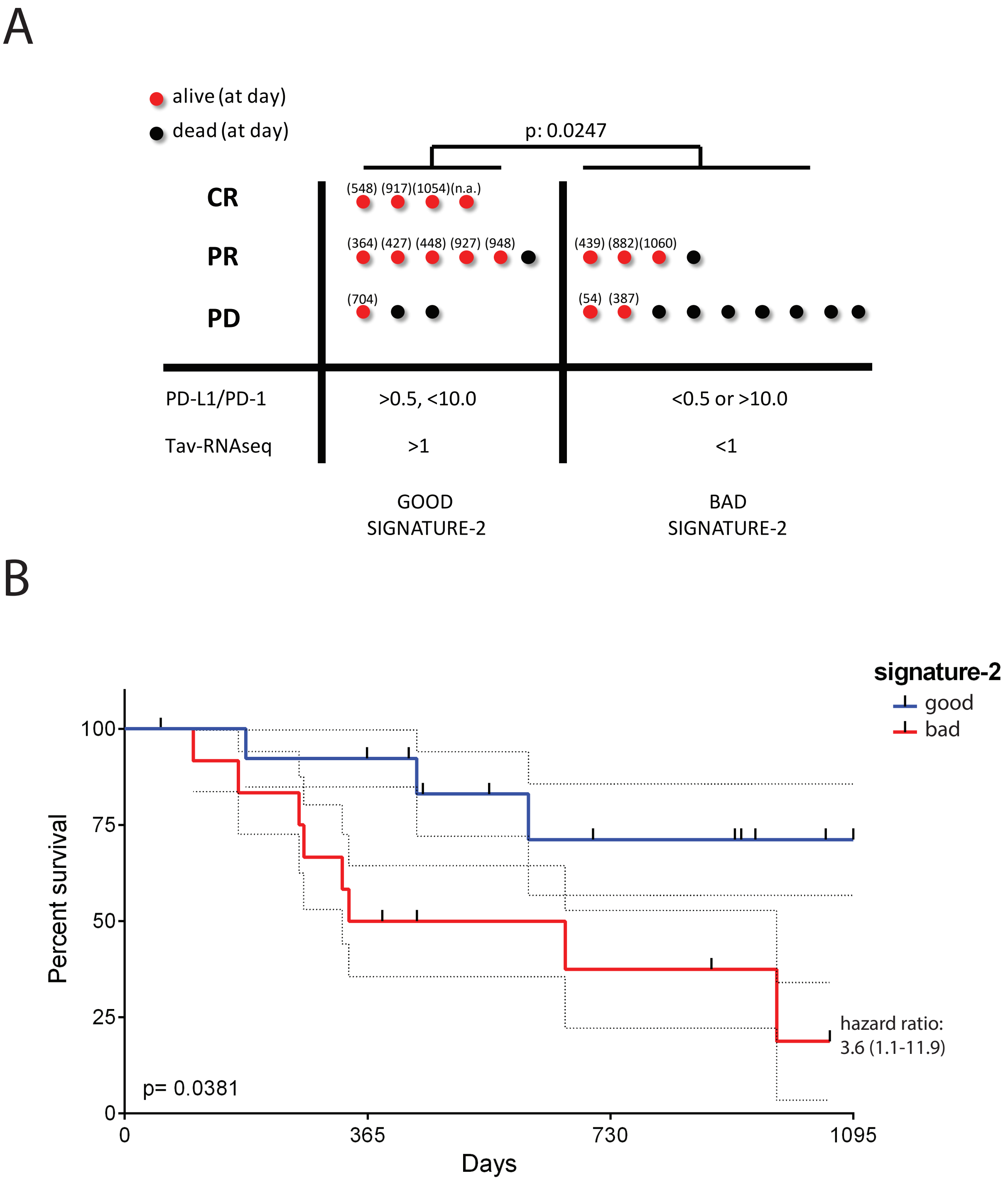

Figure S11: PD-L1/PD-1 ratio and Tav-RNAseq predict the response to nivolumab treatment

(A) The PD-L1/ PD-1 ratio and Tav-RNAseq in melanoma specimens from the study by Hugo et al. (Table S11) was evaluated, and patients were ranked into two groups: the good signature-2 group (PD-L1/PD-1 ratio between 0.5 and 10 and Tav-RNAseq greater than 1) and the bad signature-2 group (PD-L1/PD-1 ratio <0.5 or >10 and/or Tav-RNAseq lower than 1). Dots represent single patients (the red dots indicate living patients and the black dots indicate dead patients) classified according to their response to the treatment (complete response, CR; partial response, PR; progressive disease, PD). The response of the patients in the good and the bad signature-2 groups was significantly different according to the results of the contingency chi-square test.
(B) The Kaplan-Meier curve for the good signature-2 group (blue line) and the bad signature-2 group (red line) is shown. The survival of the patients in the good and the bad signature-2 groups was significantly different according to the results of the log-rank Mantel-Cox test. The hazard ratio (95%CI) was calculated with the log-rank method and is shown.
