# Supplementary material for: Identification of 15 T Cell Restricted Genes Evaluates T Cell Infiltration of Human Healthy Tissues and Cancers and Shows Prognostic and Predictive Potential
Source: Int J Mol Sci. 2019 Oct 22;20(20):5242. doi: 10.3390/ijms20205242 (PMC6829269; doi:10.3390/ijms20205242)
Supplement: Supplementary file 1 [file ijms-20-05242-s001.zip › ijms-610023-supplymentary2/Figure S2 IJMS.pdf]

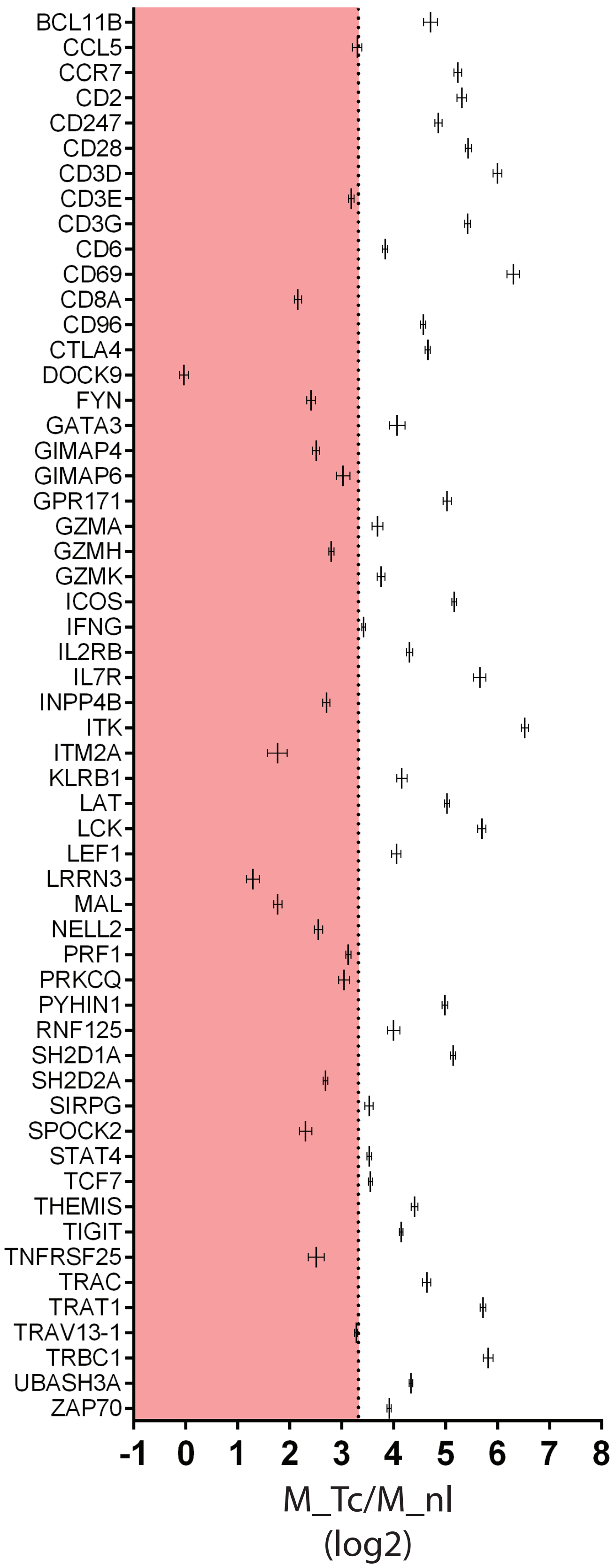

Figure S2: Exclusion of genes from the T-cell signature (round 2)

Mean gene expression by purified untouched/activated T cells (M_Tc) and mean gene expression ±SE by 152 human non-lymphoid tissues (M_nl) were expressed as log2. The difference between mean T cell expression levels and mean non-lymphoid tissue expression levels (M_Tc/M_nl) is shown as mean+1SE.
If the difference was less than 3.32log2 (red region of the graph, corresponding to a gene overexpressed by T cells by less than tenfold), the gene was excluded from the signature.
