# Supplementary material for: Identification of 15 T Cell Restricted Genes Evaluates T Cell Infiltration of Human Healthy Tissues and Cancers and Shows Prognostic and Predictive Potential
Source: Int J Mol Sci. 2019 Oct 22;20(20):5242. doi: 10.3390/ijms20205242 (PMC6829269; doi:10.3390/ijms20205242)
Supplement: Supplementary file 1 [file ijms-20-05242-s001.zip › ijms-610023-supplymentary2/Figure S3 IJMS.pdf]

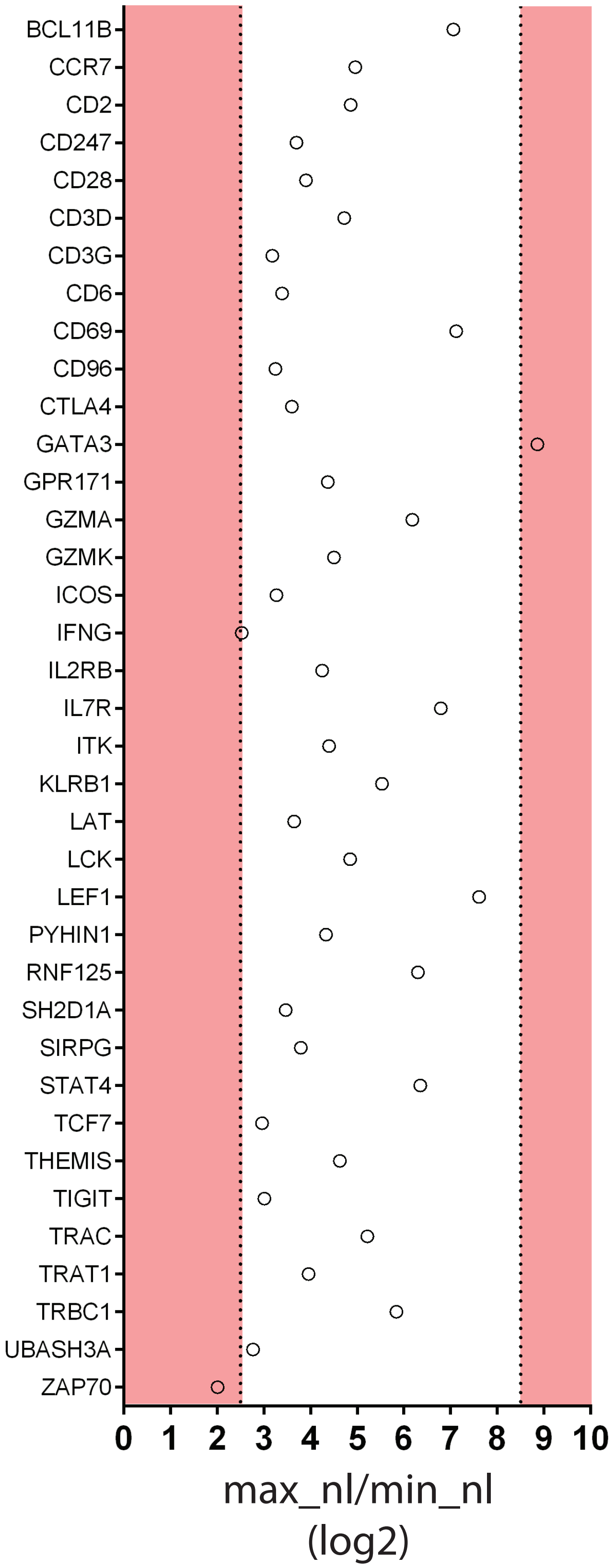


**Figure S3: Exclusion of genes from the T-cell signature (round 3)**

The expression of each gene by each of the 152 human non-lymphoid tissue (nl) was expressed as log2. Then, the difference between the maximum (max_nl) and the minimum (min_nl) expression level of each gene (max_nl/min_nl) was calculated.

We determined that for a gene to be included in the signature, the difference between the maximum and the minimum expression level of each gene (max_nl/min_nl) should be at least 2.5log2 (5.7-fold) and no more than 8.5log2 (362-fold) (a maximum range of 6log2 corresponds to a 64-fold difference between the max_nl and the min_nl). Thus, genes were excluded if their max_nl/min_nl ratio was <2.5 or >8.5log2 (red regions of the graph).
