# Supplementary material for: Identification of 15 T Cell Restricted Genes Evaluates T Cell Infiltration of Human Healthy Tissues and Cancers and Shows Prognostic and Predictive Potential
Source: Int J Mol Sci. 2019 Oct 22;20(20):5242. doi: 10.3390/ijms20205242 (PMC6829269; doi:10.3390/ijms20205242)
Supplement: Supplementary file 1 [file ijms-20-05242-s001.zip › ijms-610023-supplymentary2/Figure S4 IJMS.pdf]

Tissues:

T1	accumbens nucleus	T77	middle temporal gyrus (Brodmann area 21)
T2	adipose tissue (fat)	T78	minor salivary gland
T3	adrenal gland	T79	nasal epithelium
T4	adrenal gland cortex (adrenal cortex)	T80	nasopharyngeal (epipharyngeal) epithelium
T5	amygdala	T81	nasopharynx (epipharynx)
T6	aortic valve	T82	nipple
T7	ascending colon	T83	occipital lobe
T8	ascending colonic mucosa	T84	oral mucosa (unspecified)
T9	associative striatum	T85	ovary (female gonad)
T10	basal ganglia	T86	oviduct (fallopian tube)
T11	biceps brachii	T87	pancreas
T12	biceps femoris muscle	T88	pancreatic duct (duct of Wirsung)
T13	brain (encephalon)	T89	pancreatic islet (islet of Langerhans)
T14	breast (mammary gland)	T90	parietal lobe
T15	Brodmann area 10	T91	pars compacta
T16	Brodmann area 46	T92	pars reticulata
T17	bronchus (primary, main stem)	T93	penis
T18	caecum (cecum)	T94	pharyngeal mucosa
T19	caudate nucleus	T95	pituitary gland (hypophysis)
T20	cerebellum	T96	pons
T21	cerebral cortex (neopallium)	T97	postcentral gyrus
T22	cerebral hemisphere	T98	posterior superior temporal gyrus (Brodmann area 22)
T23	cerebral white matter	T99	prefrontal cortex
T24	choroid	T100	prostate (prostate gland)
T25	colon	T101	putamen
T26	colonic gland (crypt)	T102	pyloric antrum
T27	colonic mucosa	T103	quadriceps femoris muscle
T28	colorectum	T104	rectosigmoid colonic mucosa
T29	conjunctiva	T105	rectum
T30	cornea	T106	rectus abdominus muscle
T31	corneal limbus	T107	renal (kidney) cortex
T32	corpus callosum	T108	renal (kidney) medulla
T33	deltoid muscle	T109	renal tubulointerstitium
T34	dermal papilla	T110	retina
T35	descending colon	T111	retinal pigment epithelium
T36	descending colonic mucosa	T112	right ventricle interventricular septum
T37	diaphragm muscle	T113	salivary gland
T38	diencephalon	T114	seminal vesicle
T39	duodenum	T115	sigmoid colon
T40	entorhinal cortex	T116	sigmoid colonic mucosa
T41	epidermis	T117	skeletal muscle
T42	epididymal caput	T118	skin
T43	epididymal cauda	T119	small intestine
T44	epididymal corpus	T120	spinal cord
T45	esophagus (oesophagus)	T121	spinal ganglion (dorsal root ganglion)
T46	frontal lobe	T122	stomach
T47	frontal pole	T123	stomach cardia
T48	gall bladder	T124	stomach fundus
T49	genu	T125	subcutaneous abdominal adipose tissue (SAAT)
T50	gingiva	T126	subcutaneous adipose tissue (SAT)
T51	globus pallidus	T127	substantia nigra
T52	glomerulus	T128	subthalamic nucleus
T53	gluteal subcutaneous adipose tissue (glutSAT)	T129	superior frontal gyrus
T54	growth plate	T130	superior vestibular nucleus
T55	heart	T131	synovium (synovial membrane)
T56	heart atrium	T132	tarsal conjunctiva
T57	heart left atrial appendage	T133	temporal lobe
T58	heart left ventricle	T134	testicular lobules
T59	heart right atrial appendage	T135	testis (male gonad)
T60	heart ventricle	T136	thalamus (dorsal thalamus)
T61	hippocampus	T137	thyroid gland
T62	hypothalamus	T138	tongue
T63	ileum	T139	trachea
T64	internal (medial) globus pallidus	T140	transverse colon
T65	jejunum	T141	trigeminal ganglion
T66	joint	T142	urethra
T67	joint synovium	T143	urinary bladder
T68	kidney	T144	uterine cervix
T69	labial salivary gland	T145	uterine endometrium
T70	large intestine	T146	uterine myometrium
T71	lateral thalamic nucleus	T147	uterus
T72	liver	T148	vagina
T73	lung	T149	vastus lateralis muscle
T74	medial rectus muscle	T150	ventral tegmental area
T75	medulla oblongata	T151	vermis
T76	midbrain (mesencephalon)	T152	vulva

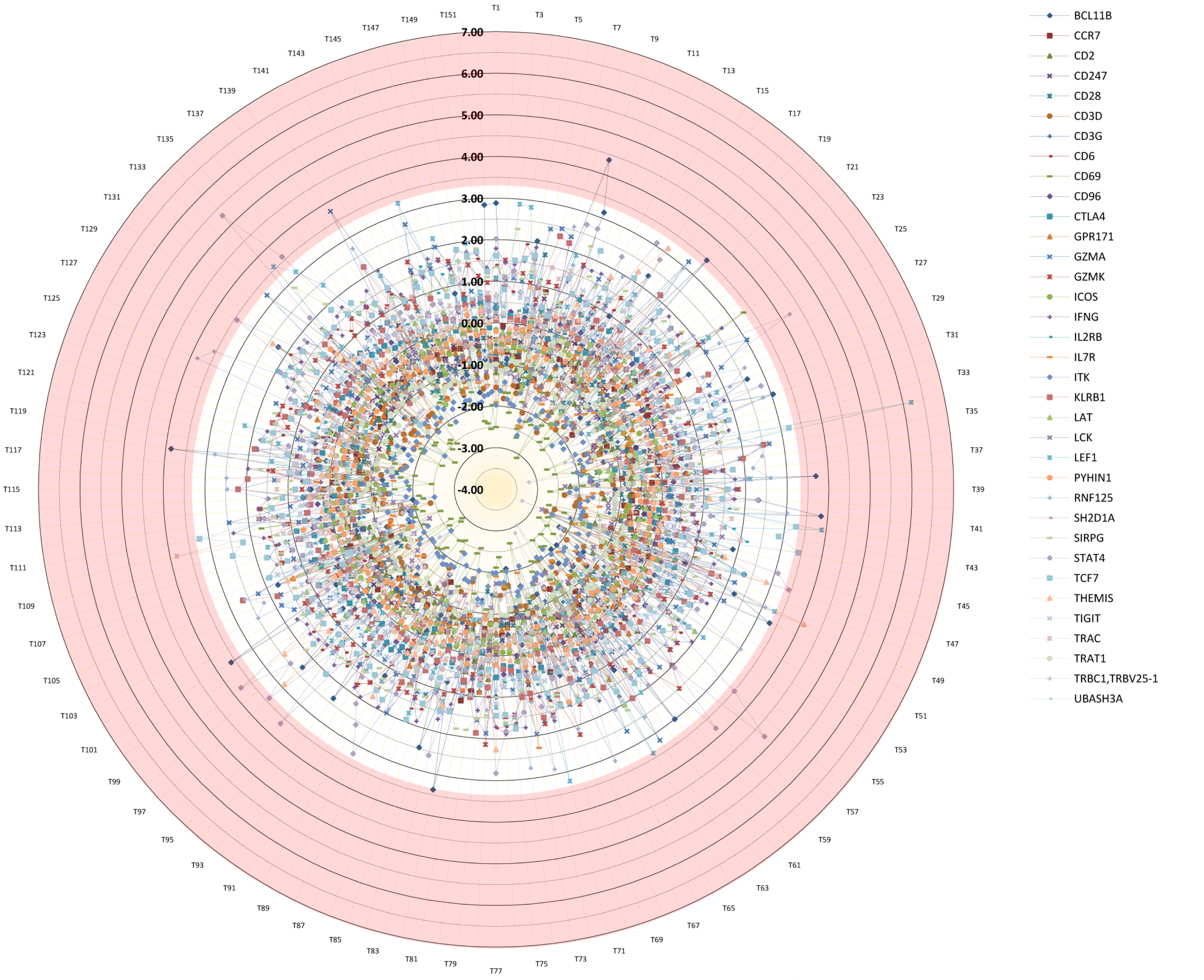

Figure S4: Exclusion of genes from the T-cell signature (round 4)

The expression of each gene by each of the 152 human non-lymphoid tissue (nl) and the mean expression of each gene by purified untouched/activated T cells (Tc) were expressed as log2. Then, the difference between expression in each non-lymphoid tissue and mean T-cell expression (nl/Tc) was evaluated, and the mean of nl/Tc (M_nl/Tc) was calculated for each tissue.

The cut-off for the difference between nl/Tc and M_nl/Tc ([nl/Tc]/[M_nl/Tc]) of each gene was set as 3.32log2 (representing a tenfold difference). If the (nl/Tc)/(M_nl/Tc) value for a gene was more than 3.32log2 (red region of the graph) for at least for two tissues (we chose two instead of one tissue to avoid technical artifacts or peculiarity concerning just one tissue), it was excluded.
