# Supplementary material for: Identification of 15 T Cell Restricted Genes Evaluates T Cell Infiltration of Human Healthy Tissues and Cancers and Shows Prognostic and Predictive Potential
Source: Int J Mol Sci. 2019 Oct 22;20(20):5242. doi: 10.3390/ijms20205242 (PMC6829269; doi:10.3390/ijms20205242)
Supplement: Supplementary file 1 [file ijms-20-05242-s001.zip › ijms-610023-supplymentary2/Figure S5 IJMS.pdf]

Cell Lines:

L1	5637	L159	Hep-G2	L317	NCI-H630
L2	1A2	L160	Hep-G2/C3A	L318	NCI-H647
L3	22Rv1	L161	HGC-27	L319	NCI-H650
L4	23132/87	L162	HK-2 [Human kidney]	L320	NCI-H661
L5	293/pmiR-155	L163	HL-60	L321	NCI-H69
L6	293/pmiR-K12-11	L164	HMCB	L322	NCI-H716
L7	639V	L165	HOP-62	L323	NCI-H720
L8	647V	L166	HOP-92	L324	NCI-H747
L9	769-P	L167	HOS	L325	NCI-H748
L10	76N-RHOA	L168	HPAC	L326	NCI-H810
L11	76N-RHOA MUT p.G14V	L169	HPAF-II	L327	NCI-H82
L12	76N-RHOA MUT p.T37A	L170	Hs 294T	L328	NCI-H838
L13	786-O	L171	Hs 578T	L329	NCI-N87
L14	A101D	L172	Hs 746.T	L330	neuroblastoma
L15	A-172	L173	Hs 766T	L331	NPrEC
L16	A-204	L174	Hs 840.T	L332	NUGC-3
L17	A2780	L175	HS-Sultan	L333	NUGC-4
L18	A-375	L176	HT	L334	NuLi-1
L19	A-427	L177	HT-1080	L335	OAW42
L20	A-431	L178	HT-1197	L336	OCI-AML5
L21	A-498	L179	HT-1376	L337	OE19
L22	A-549	L180	HT-29	L338	OE21
L23	A-673	L181	HT29-R	L339	OE33
L24	ACHN	L182	HT-3	L340	OV-90
L25	AGS	L183	HuNS1	L341	OVCA429
L26	AN3-CA	L184	HuP-T3	L342	OVCAR-3
L27	ARH-77	L185	HUP-T4	L343	OVCAR-4
L28	ARPE-19	L186	IGROV-1	L344	OVCAR-5
L29	AsPC-1	L187	IGROV-1-con	L345	OVCAR-8
L30	AU565	L188	IGROV-1-CXCR4 KD	L346	OVCAR-8 RIA
L31	AZ-521	L189	IM95	L347	OVCAR-8 RIIB
L32	BC-1	L190	IMR-32	L348	P3HR-1
L33	BC-2	L191	J82	L349	p53HCT116
L34	BC-3	L192	JAR	L350	Panc 02.03
L35	BDCM	L193	JEG-3	L351	Panc 03.27
L36	BEAS-2B	L194	Jiyoye	L352	Panc 04.03
L37	BeWo	L195	JM-1	L353	Panc 08.13
L38	BFTC-905	L196	JVM-3	L354	Panc 10.05
L39	BHT-101	L197	K-562	L355	PANC-1
L40	BM-1604	L198	Kasumi-2	L356	PC-3
L41	BT-20	L199	KATO III	L357	PE/CA-PJ15
L42	BT-474	L200	KG-1	L358	PE/CA-PJ34 (clone C12)
L43	BT-549	L201	KHOS-240S	L359	PE/CA-PJ41 (clone D2)
L44	BV-173	L202	KLE	L360	PE/CA-PJ49
L45	BxPC-3	L203	KM12	L361	PLB-985
L46	C32 [Human melanoma]	L204	KPL-1	L362	PNET
L47	C32TG	L205	Ku812	L363	PSN1
L48	C-33 A	L206	KYSE-30	L364	Raji
L49	C-4 I	L207	L3.6pl	L365	RCH-ACV
L50	C-4 II	L208	L-428	L366	RD
L51	CA46	L209	LL24	L367	RD-ES
L52	Caco-2	L210	LN-18	L368	REC-1
L53	CA-HPV-10	L211	LNCaP	L369	Reh
L54	Caki-1	L212	LoVo	L370	Rh30
L55	Caki-2	L213	LOX-IMVI	L371	RKO
L56	CAL-27	L214	LS1034	L372	RKO-E6
L57	CAL-54	L215	LS123	L373	RL
L58	CAL-62	L216	LS174T	L374	RL95-2
L59	CAL-85-1	L217	LS180	L375	RPMI 6666
L60	Calu-1	L218	M14	L376	RPMI-8226
L61	Calu-3	L219	M2A7	L377	RT-112
L62	Calu-6	L220	Malme-3M	L378	RWPE-1
L63	CAMA-1	L221	MC/CAR	L379	RXF 393L
L64	Caov-3	L222	MC116	L380	SCaBER
L65	Capan-1	L223	MCF-10A	L381	SEM
L66	Capan-2	L224	MCF-7	L382	SF268
L67	CCD-1079Sk	L225	MCF-7.5C	L383	SF295
L68	CCF-STTG1	L226	MCF-7:2A	L384	SF539
L69	CFPAC-1	L227	MCF-7-con	L385	SH-4
L70	CGTH-W-1	L228	MCF-7-MT2A-MYC	L386	SHP-77
L71	ChaGo-K-1	L229	MCF-7-MT2A-Myc MUT p.40-178del	L387	SH-SY5Y
L72	CHL-1	L230	MDA-MB-157	L388	SiHa
L73	CHP-212	L231	MDA-MB-175-VII	L389	SJSA-1
L74	COLO 201	L232	MDA-MB-231	L390	SK-BR-3
L75	COLO 205	L233	MDA-MB-361	L391	SK-CO-1
L76	COLO 320DM	L234	MDA-MB-415	L392	SK-LMS-1
L77	COLO 320HSR	L235	MDA-MB-435S	L393	SK-MEL-1
L78	COLO 357/FG	L236	MDA-MB-436	L394	SK-MEL-2
L79	COLO 668	L237	MDA-MB-453	L395	SK-MEL-28
L80	COLO 678	L238	MDA-MB-468	L396	SK-MEL-3
L81	COLO 704	L239	MEC-1	L397	SK-MEL-5
L82	COLO 741	L240	MEG-01	L398	SK-MES-1
L83	COLO 829	L241	MES-SA	L399	SK-N-AS
L84	COR-L105	L242	MFE-296	L400	SK-N-BE(2)-C
L85	COR-L23	L243	MG-63	L401	SK-N-DZ
L86	COR-L279	L244	MHH-PREB-1	L402	SK-NEP-1
L87	COR-L88	L245	MIA PaCa-2	L403	SK-N-FI
L88	CP-A	L246	MKN1	L404	SK-N-MC
L89	CRO-AP2	L247	MKN45	L405	SKO-007
L90	CRO-AP5	L248	MKN74	L406	SK-OV-3
L91	CuFi-1	L249	ML-2	L407	SK-UT-1
L92	D283 Med	L250	MM1.S	L408	SN12C
L93	D341 Med	L251	MT3	L409	SNB-19
L94	Daoy	L252	MV4-11	L410	SNB-75
L95	Daoy-ALK KD	L253	MX-1	L411	SNU-1
L96	Daoy-BMI1 KD	L254	Namalwa	L412	SNU-16
L97	Daoy-BMI1 KD-PCGF2 KD	L255	NB4	L413	SNU-182
L98	Daoy-PCGF2 KD	L256	NC-37	L414	SNU-387
L99	Daudi	L257	NCI-ADR-RES	L415	SNU-398
L100	DB	L258	NCI-H1048	L416	SNU-423
L101	DBTRG-05MG	L259	NCI-H1092	L417	SNU-449
L102	Detroit 562	L260	NCI-H1155	L418	SNU-475
L103	DG-75	L261	NCI-H1299	L419	SNU-5
L104	DK-MG	L262	NCI-H1299-rTetR-TP63	L420	SQ-20B
L105	DMS 114	L263	NCI-H1299-rTetR-TP63 MUT p.Q540L	L421	ST486
L106	DMS 153	L264	NCI-H1355	L422	ST88-14
L107	DMS 273	L265	NCI-H1395	L423	SU-DHL-10
L108	DMS 53	L266	NCI-H1436	L424	SU-DHL-16
L109	DMS 79	L267	NCI-H1437	L425	SU-DHL-5
L110	DoHH2	L268	NCI-H1563	L426	SU-DHL-6
L111	DoTc2 4510	L269	NCI-H1573	L427	SW1088
L112	DU145	L270	NCI-H1581	L428	SW1116
L113	DU4475	L271	NCI-H1618	L429	SW1353
L114	EB1	L272	NCI-H1623	L430	SW1417
L115	EB2	L273	NCI-H1650	L431	SW1463
L116	EB3	L274	NCI-H1651	L432	SW1573
L117	EFM-19	L275	NCI-H1666	L433	SW1783
L118	EKVX	L276	NCI-H1694	L434	SW1990
L119	ES-2	L277	NCI-H1703	L435	SW403
L120	FaDu	L278	NCI-H1770	L436	SW48
L121	G-401	L279	NCI-H1792	L437	SW480
L122	G-402	L280	NCI-H1793	L438	SW480-con
L123	GA-10	L281	NCI-H1838	L439	SW620
L124	GCT	L282	NCI-H1869	L440	SW684
L125	GDM-1	L283	NCI-H187	L441	SW756
L126	GM05659	L284	NCI-H1930	L442	SW780
L127	GTL-16	L285	NCI-H1975	L443	SW837
L128	H4	L286	NCI-H1993	L444	SW872
L129	HAECT	L287	NCI-H2009	L445	SW900
L130	HCC1143	L288	NCI-H2030	L446	SW948
L131	HCC1187	L289	NCI-H2052	L447	SW954
L132	HCC1395	L290	NCI-H2081	L448	SW982
L133	HCC1419	L291	NCI-H2087	L449	T3M-4
L134	HCC1569	L292	NCI-H209	L450	T-47D
L135	HCC1599	L293	NCI-H2107	L451	T84
L136	HCC1806	L294	NCI-H2122	L452	Tanoue
L137	HCC1937	L295	NCI-H2126	L453	THP-1
L138	HCC1954	L296	NCI-H2170	L454	TK-10
L139	HCC202	L297	NCI-H2171	L455	Toledo
L140	HCC2157	L298	NCI-H2195	L456	T-Rex-293
L141	HCC2218	L299	NCI-H2228	L457	TT
L142	HCC2998	L300	NCI-H226	L458	U-251MG
L143	HCC38	L301	NCI-H23	L459	U266B1
L144	HCC70	L302	NCI-H2347	L460	U-87MG
L145	HCC827	L303	NCI-H2405	L461	U-937
L146	HCT 116	L304	NCI-H292	L462	UACC-257
L147	HCT 116 DICER1(-/-)	L305	NCI-H295R	L463	UACC-62
L148	HCT 15	L306	NCI-H322	L464	UACC-812
L149	HCT 8	L307	NCI-H358	L465	UACC-893
L150	HD-MY-Z	L308	NCI-H441	L466	UMC-11
L151	HEC-1-A	L309	NCI-H446	L467	UM-UC-3
L152	HEC-1-B	L310	NCI-H460	L468	UO-31
L153	HEK293	L311	NCI-H508	L469	WI-38
L154	HEK293-con	L312	NCI-H520	L470	WiDr
L155	HEK-TLR2	L313	NCI-H522	L471	WM115
L156	HEL 92.1.7	L314	NCI-H524	L472	Y-79
L157	HeLa	L315	NCI-H526	L473	YAPC
L158	Hep 3B2.1-7	L316	NCI-H596	L474	ZR-75-30

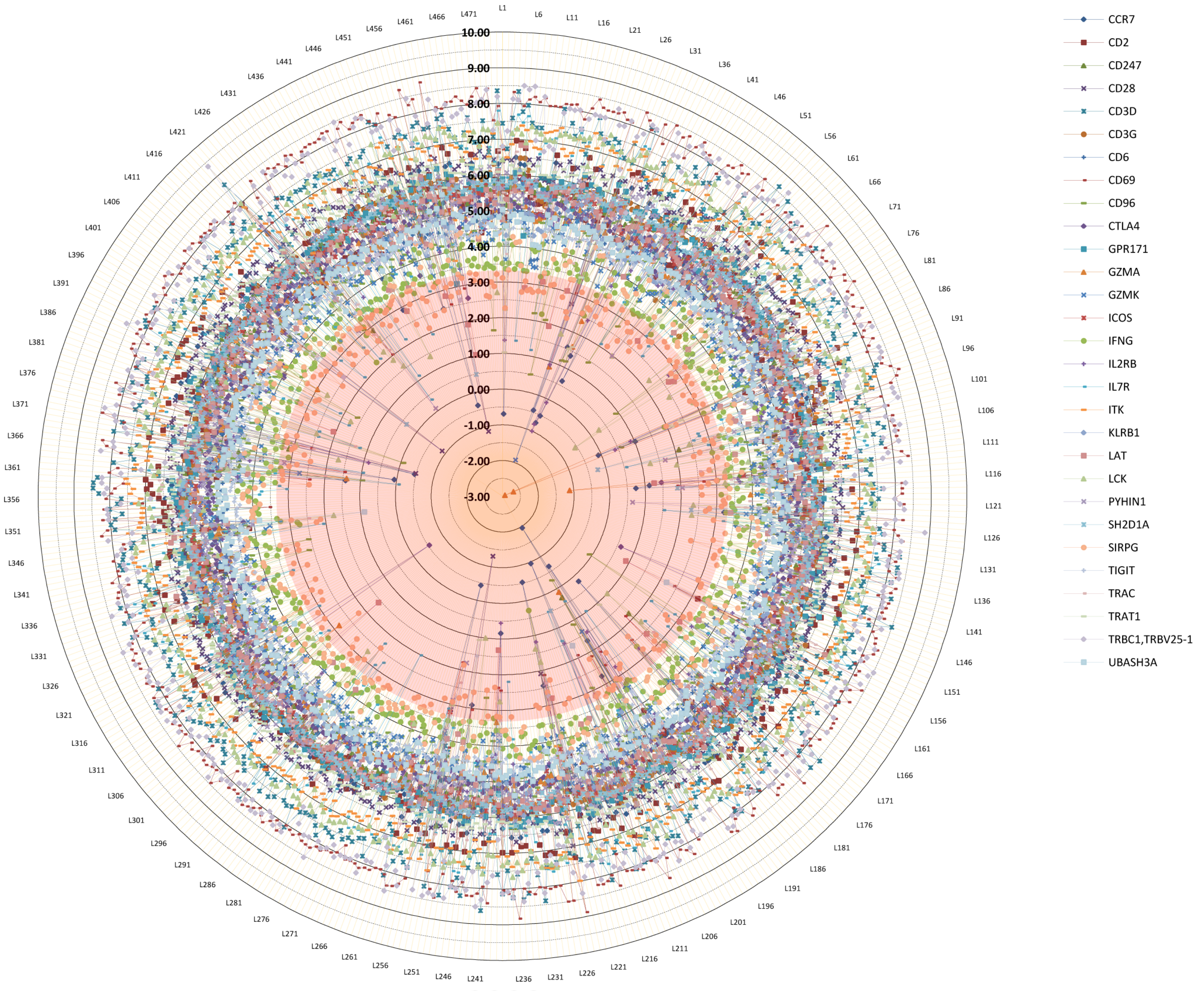

Figure S5: Exclusion of genes from the T-cell signature (round 6)

To evaluate if the selected genes were overexpressed by activated parenchymal cells (for example, by cancer cells) and activated non-T immune cells, we tested the expression of genes still present in the developing signature in cell lines derived from non-T cells and tissues. The mean expression of each gene by purified untouched/activated T cells (Tc) and the expression of each gene by each human non-T cell line (nTcl) was expressed as log2. Then, the difference between the mean expression of each gene by T cells and its expression in each human non-T cell line (Tc/nTcl) was calculated for each cell line. If the difference was less than 3.32log2 (representing less than tenfold overexpression, red region of the graph) in cell lines from at least two origins (we chose two instead of one tissue to avoid technical artifacts or peculiarity concerning just one tissue), the gene was excluded.
