# Supplementary material for: Identification of 15 T Cell Restricted Genes Evaluates T Cell Infiltration of Human Healthy Tissues and Cancers and Shows Prognostic and Predictive Potential
Source: Int J Mol Sci. 2019 Oct 22;20(20):5242. doi: 10.3390/ijms20205242 (PMC6829269; doi:10.3390/ijms20205242)
Supplement: Supplementary file 1 [file ijms-20-05242-s001.zip › ijms-610023-supplymentary2/Figure S6 IJMS.pdf]

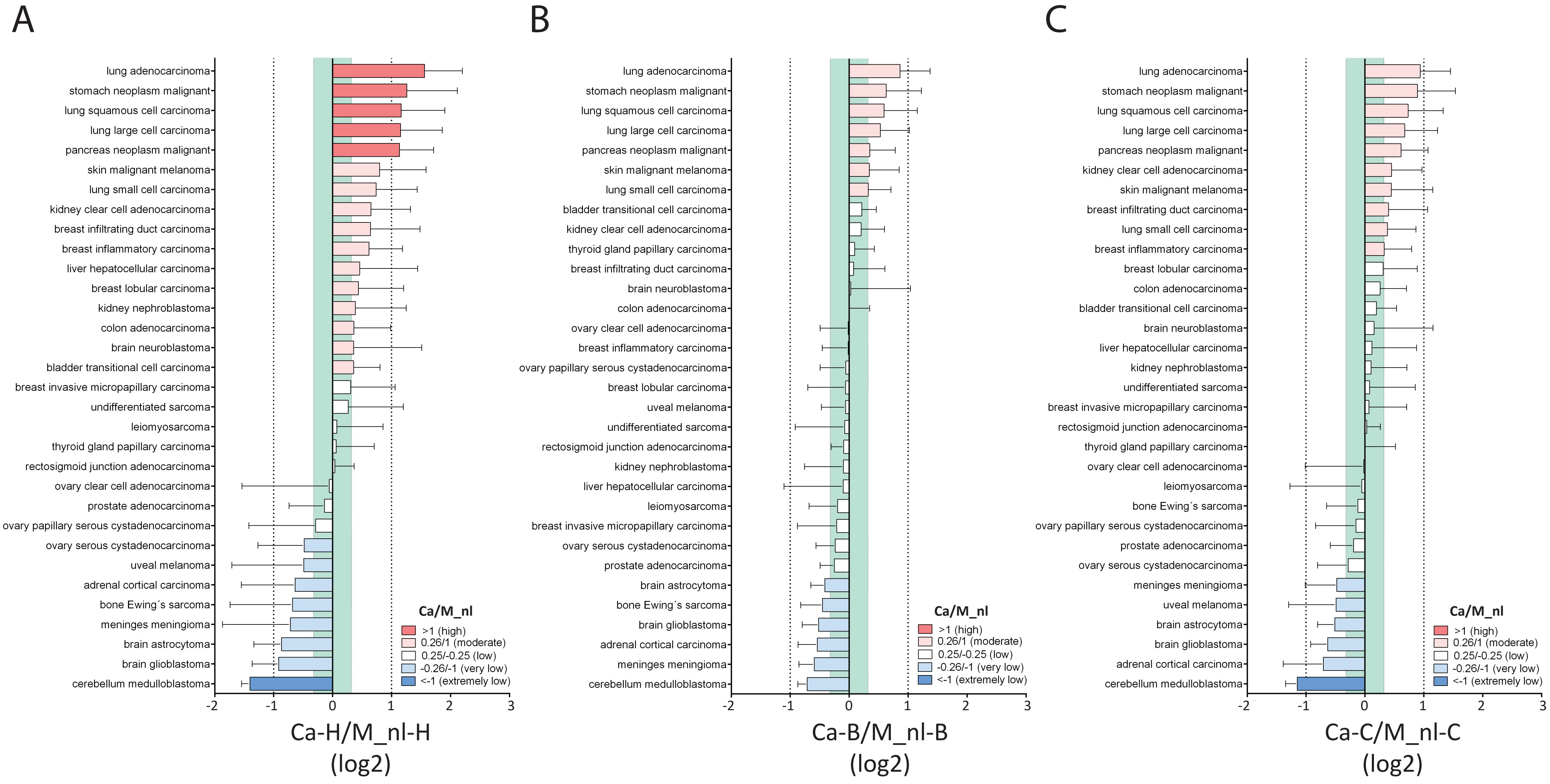

Figure S6: Ts% of 32 types of cancers evaluated using signatures H, B, and C

The mean±1SD expression of the genes in signature-H (panel A), signature-B (panel B), and signature-C (panel C) in 32 types of cancers (Ca_H, Ca_B, Ca_C) and the mean expression of the genes in non-lymphoid tissues (M_nl-H, M_nl-B, M_nl-C) were calculated. Then, the difference between the mean±1SD expression of the signature genes in each cancer and the mean expression of the genes in non-lymphoid tissues (Ca_H/M_nl-H, Ca_B/M_nl-B, Ca_C/M_nl-C) was calculated.
If a cancer specimen showed a Ca/M_nl value between 0.25 and -0.25log2 (that is a T cell expression level between 80% and 125% of M_nl)(green area), it was considered to have a similar low infiltration level as the corresponding non-lymphoid tissue (white bars). If a cancer showed a Ca/M_nl value between 0.26 and 1log2 (that is a T cell expression level between 125% and 200% of M_nl), it was considered to be infiltrated by T cells at moderate levels (pink bars). If a cancer showed a value Ca/M_nl above 1log2 (that is a T cell expression level above 200% of M_nl), it was considered to be infiltrated by T cells at high levels (red bars). If a cancer showed a Ca/M_nl value between -1 and -0.26log2 (that is a T cell expression level between 50% and 80% of M_nl), it was considered to be infiltrated by T cells at very low levels (light blue bars). If a cancer showed a Ca/M_nl value below -1log2 (that is a T cell expression level below 50% of M_nl), it was considered to be infiltrated by T cells at extremely low levels (blue bars).
