# Supplementary material for: Identification of 15 T Cell Restricted Genes Evaluates T Cell Infiltration of Human Healthy Tissues and Cancers and Shows Prognostic and Predictive Potential
Source: Int J Mol Sci. 2019 Oct 22;20(20):5242. doi: 10.3390/ijms20205242 (PMC6829269; doi:10.3390/ijms20205242)
Supplement: Supplementary file 1 [file ijms-20-05242-s001.zip › ijms-610023-supplymentary2/Figure S7 IJMS.pdf]

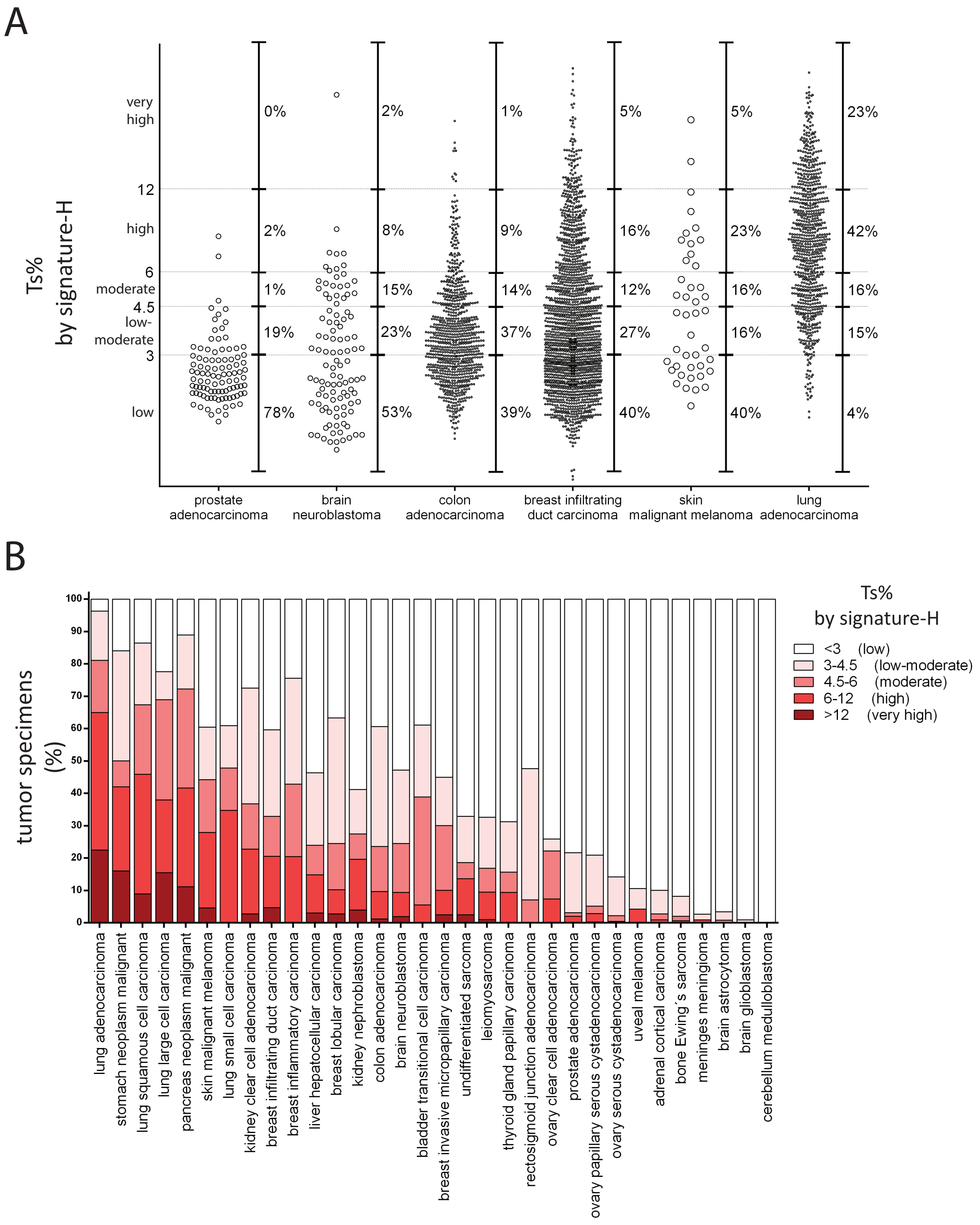

Figure S7. Tci score % of cancer specimens from patients evaluated using signature-H

(A) Tci score % (Ts%) of cancer specimens from patients for six types of cancers. The expression of genes of signature-H in 32 types of cancers in each cancer specimen was divided by the mean expression of the genes in T cells, and the expression of genes of signature-H was expressed as a percentage. The percentage of specimens with low, low-moderate, moderate, high, and very high Ts% is reported for each cancer and the percentage of patients in each Ts% category is shown on the right.

(B) The percentage of specimens with low, low-moderate, moderate, high, and very high Ts% according to signature-H is reported for 32 types of cancer.
