# Supplementary material for: Identification of 15 T Cell Restricted Genes Evaluates T Cell Infiltration of Human Healthy Tissues and Cancers and Shows Prognostic and Predictive Potential
Source: Int J Mol Sci. 2019 Oct 22;20(20):5242. doi: 10.3390/ijms20205242 (PMC6829269; doi:10.3390/ijms20205242)
Supplement: Supplementary file 1 [file ijms-20-05242-s001.zip › ijms-610023-supplymentary2/Figure S8 IJMS.pdf]

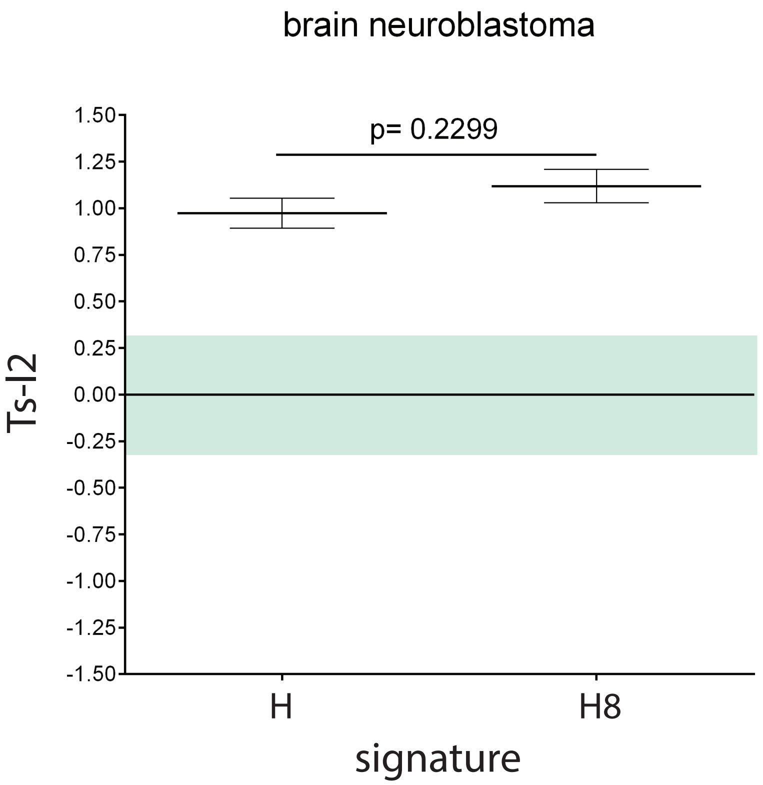

Figure S8: Similar performance of Signature-H8 and signature-H for the evaluation of the Ts-I2 of brain neuroblastoma

The Ts-I2 of brain neuroblastoma specimens was calculated using the genes included in signatures H and 8 genes included in signature H that are present in the Agilent custom array A-MEXP-1746 (signature-H8).
The Ts-I2 of brain neuroblastoma was calculated as the difference between the mean expression (log2) of the signature genes in the cancer specimens and the mean expression (log2) of the same genes in the corresponding HT (brain). The mean Ts-I2±1SE is reported. A Ts-I2 between 0.32 and -0.32 (green area) was considered to indicate that the Tci of the tumor was similar to that of the corresponding HT. Student's *t*-test was used for comparing the Ts-I2 calculated using signature-H and the Ts-I2 calculated using signature H8.
