# Supplementary material for: Identification of 15 T Cell Restricted Genes Evaluates T Cell Infiltration of Human Healthy Tissues and Cancers and Shows Prognostic and Predictive Potential
Source: Int J Mol Sci. 2019 Oct 22;20(20):5242. doi: 10.3390/ijms20205242 (PMC6829269; doi:10.3390/ijms20205242)
Supplement: Supplementary file 1 [file ijms-20-05242-s001.zip › ijms-610023-supplymentary2/Figure S9 IJMS.pdf]

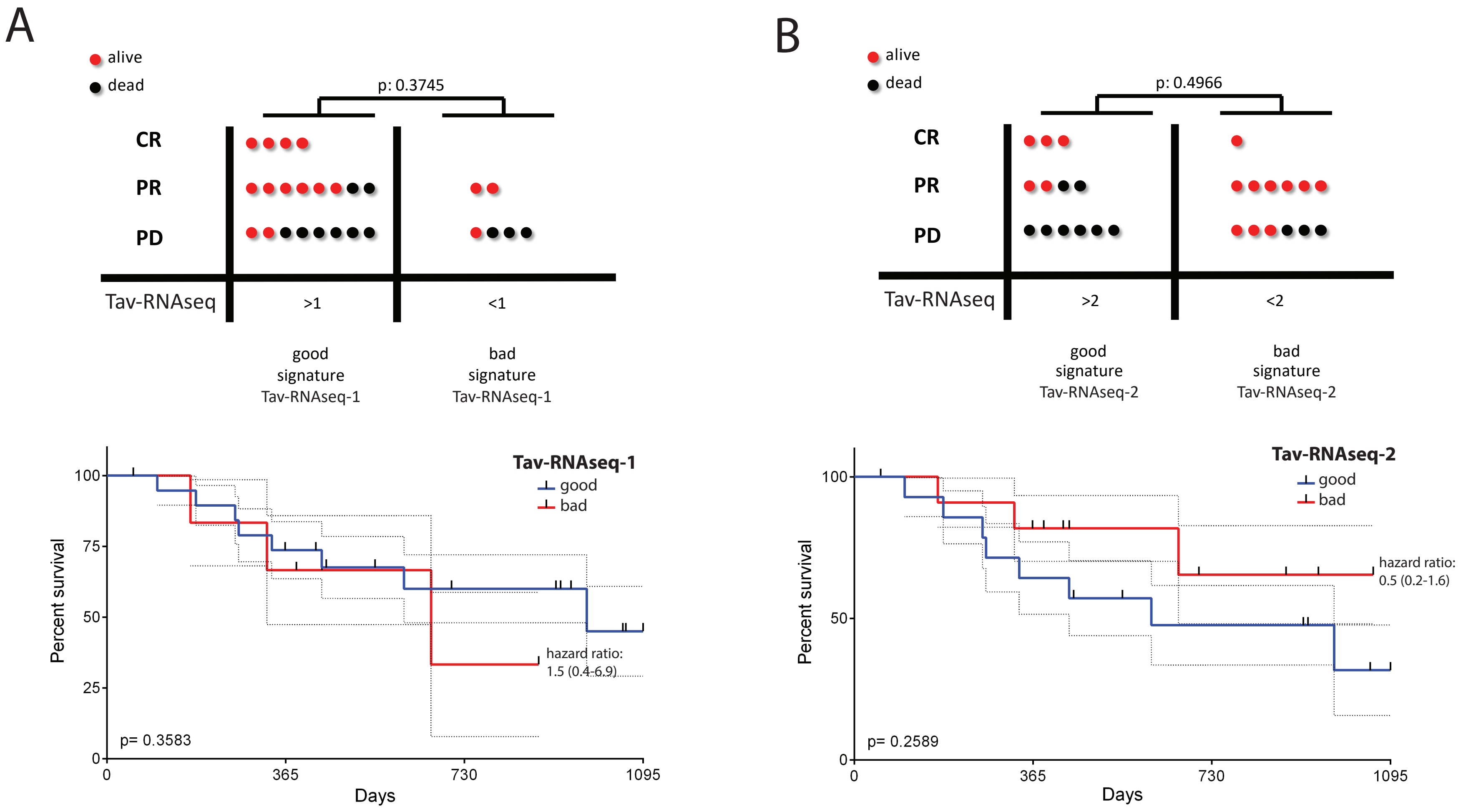

Figure S9: No correlation between T-cell infiltration and response to nivolumab

(A) (top panel): The mean expression level of signature-H genes in melanoma specimens (Tav-RNAseq)(Table S11) was evaluated, and the patients were accordingly divided into the good signature Tav-RNAseq-1 (specimens with Tav-RNAseq>1) and the bad signature Tav-RNAseq-1 groups (specimens with Tav-RNAseq<1). Dots represent single patients (the red dots indicate living patients and the black dots indicate dead patients) classified according to their response to the treatment (complete response, CR; partial response, PR; progressive disease, PD). The response of the patients in the good and the bad signature Tav-RNAseq-1 was not significantly different according to the results of the contingency chi-square test.

(A) (bottom panel): The Kaplan-Meier curve for the good signature Tav-RNAseq-1 group (blue line) and the bad signature Tav-RNAseq-1 group (red line) is shown. The survival of the patients in the good and the bad signature Tav-RNAseq-1 groups was not significantly different according to the results of the log-rank Mantel-Cox test. The hazard ratio (95%CI) was calculated with the log-rank method and is shown.

(B) (top panel): The mean expression level of signature-H genes in melanoma specimens (Tav-RNAseq)(Table S11) was evaluated, and the patients were accordingly divided into the good signature Tav-RNAseq-2 (specimens with Tav-RNAseq>2) and the bad signature Tav-RNAseq-2 groups (specimens with Tav-RNAseq<2). Dots represent single patients (the red dots indicate living patients and the black dots indicate dead patients) classified according to their response to the treatment (complete response, CR; partial response, PR; progressive disease, PD). The response of the patients in the good and the bad signature Tav-RNAseq-2 groups was not significantly different according to the results of the contingency chi-square test.

(B) (bottom panel): The Kaplan-Meier curve for the good signature Tav-RNAseq-2 group (blue line) and the bad signature Tav-RNAseq-2 group (red line) is shown. The survival of the patients in the good and the bad signature Tav-RNAseq-2 groups was not significantly different according to the results of the log-rank Mantel-Cox test. The hazard ratio (95%CI) was calculated with the log-rank method and is shown.
